# Urinary complement factor D is increased in primary malignant hypertension: a single-center, cross-sectional study

**DOI:** 10.1038/s41598-024-66875-4

**Published:** 2024-07-15

**Authors:** Yaqi Cheng, Weiwei Qin, Liling Lin, Youhe Gao, Mingxi Li

**Affiliations:** 1 Department of Nephrology, State Key Laboratory of Complex Severe and Rare Diseases, Peking Union Medical College Hospital, Peking Union Medical College and Chinese Academy of Medical Sciences, Beijing, 100730 China; 2 Department of Anesthesiology, Qingdao Hospital, University of Health and Rehabilitation Sciences (Qingdao Municipal Hospital), Qingdao, 266071 China; 3 Department of Biochemistry and Molecular Biology, Gene Engineering Drug and Biotechnology Beijing Key Laboratory, Beijing Normal University, Beijing, 100875 China; 4 Department of Laboratory, State Key Laboratory of Complex Severe and Rare Diseases, Peking Union Medical College Hospital, Peking Union Medical College and Chinese Academy of Medical Sciences, Beijing, 100730 China

**Keywords:** Complement factor D, Malignant hypertension, Urinary biomarker, Diagnostic markers, Hypertension, Glomerular diseases

## Abstract

Kidney injury is one of the detrimental consequences of primary malignant hypertension (pMHTN). There is a paucity of non-invasive biomarkers to enhance diagnosis and elucidate the underlying mechanisms. This study aims to explore urine protein biomarkers for pMHTN associated renal damage. In the discovery phase, urine samples were collected from 8 pMHTN, 19 disease controls (DCs), and 5 healthy controls (HCs). In-gel digestion combined with liquid chromatography–tandem mass spectrometry (LC–MS/MS) approach was used for identification of proteins associated with pMHTN. In the validation phase, the differentially expressed proteins were validated by ELISA assay in cohort with 10 pMHTN patients, 37 DCs, and 30 HCs. Compared to DCs and HCs, a specific band between 15 and 25 kDa was found in 7 out of 8 patients with pMHTN. Further LC–MS/MS analysis revealed 5 differentially expressed proteins. ELISA validation demonstrated that urinary complement factor D (CFD) was significantly up regulated in pMHTN. By receiver operating characteristic curve analysis, urinary CFD/Cr showed moderate potential in discriminating pMHTN from DCs (the area under curve: 0.822, 95% CI 0.618–0.962). Urinary CFD may be a potential biomarker for pMHTN with its elevation indicative of the activation of the alternative complement pathway in pMHTN.

## Introduction

Malignant hypertension (MHTN) is a hypertensive emergency characterized by severe blood pressure elevation (typically ≥ 200/120 mmHg) accompanied by bilateral retinal hemorrhage and/or papilledema, along with progressive multi-organ damage^1^. The distinctive vascular features in MHTN consists of hyperplastic arteriosclerosis and fibrinoid necrosis, which falls under the category of thrombotic microangiopathy (TMA)^2,3^. Despite significant improvements in the survival rates of patients with MHTN following the development of novel antihypertensive medications, renal prognosis varies when MHTN is complicated by renal damage, with 5-year renal survival rates ranging from 47% to 90%^4–6^. Therefore, further investigation into the pathophysiological mechanisms underlying MHTN is of prominent clinical relevance.

Primary malignant hypertension (pMHTN) accounts for approximately 20% to 60% of MHTN, with its pathogenic mechanisms yet to be elucidated^3,7^. The diagnosis requires the exclusion of secondary hypertension. In cases where glomerulonephritis is suspected, renal biopsy remains the sole means of diagnosis^3,8^. Consequently, there is an urgent need for non-invasive biomarkers to diagnose pMHTN at an early stage, thereby allowing timely therapeutic interventions^5^.

Urine is a promising resource for biomarker studies^9^. Our previous studies have identified more than 8000 proteins in human urine, 40% of which are derived from plasma proteins^9^. Urine has been demonstrated to reflect early changes during the onset and progression of various diseases, such as diabetic nephropathy^10^, bladder cancer^11^, and colorectal carcinoma^12^. For certain conditions, urine may serve as a more sensitive and specific biomarker source than blood^13,14^. Proteomic analysis of urine has the potential to identify non-invasive biomarkers for pMHTN.

In the present study, we initially performed urinary sodium sodecy sulfate polyacrylamide gel electrophoresis (SDS-PAGE) of patients with various diseases and observed distinct protein bands from pMHTN patients compared to the control groups. Further qualitative and quantitative analyses of the differential proteins were conducted using in-gel digestion followed by two-dimensional liquid chromatography-tandem mass spectrometry (LC–MS/MS), revealing specific urinary proteins associated with pMHTN. Subsequently, enzyme-linked immunosorbent assay (ELISA) validation in an independent cohort verified urinary complement factor D (CFD) as a noninvasive diagnostic biomarker for pMHTN. These findings contribute novel insights into the noninvasive diagnosis of pMHTN and complement-mediated pathogenesis of the disease.

## Result

### Discovery of differential proteins through SDS-PAGE analysis

The discovery phase incorporated 8 patients with pMHTN, 11 renal disease controls (DCs) (including 3 IgA nephropathy (IgAN), 3 membranous nephropathy (MN), 2 lupus nephritis (LN), and 3 IgA vasculitis), 8 non-renal DCs (4 with diabetes, 2 with hypertension, and 2 with Behcet's disease), and 5 healthy controls (HCs) (Table 1). All eight pMHTN patients showed no signs of microangiopathic hemolytic anemia or thrombocytopenia. Blood pressure in pMHTN patients was significantly higher compared to DCs (p < 0.001). The eGFR was significantly lower in the pMHTN group compared to DCs (26.32 ± 10.94 vs 102.1 ± 25.38 mL/min/1.73 m^2^, p < 0.001). No significant differences were observed between the pMHTN group and DCs regarding age, gender, hemoglobin, platelets, LDH, and other parameters (Supplementary Table 1).

**Table 1:** Clinical features and laboratory findings of patients in the discovery phase.

Number	Gender/Age	Diagnosis	BP, mmHg	Hypertensive retinopathy	eGFR, mL/min/1.73 m^2^	Hb, g/L	Plt, × 10^9^/L	C3, g/L	C4, g/L	LDH, U/L	24 h UP, g	Antihypertensive drugs
pMHTN												
1	M/32	MHTN	200/140	Grade 3	40.35	149	189	1.11	0.24	182	1.7	CCB/ARB/β-blocker/α-blocker
2	M/46	MHTN	213/130	Grade 3	33.5	120	272	1.39	0.54	207	0.85	CCB/ACEI/β-blocker
3	M/39	MHTN	220/145	Grade 3	15.64	143	237	0.93	0.32	165	1.1	CCB/β-blocker/α-blocker/diuretics
4	M/30	MHTN	230/150	Grade 3	20.61	148	259	1.17	0.35	176	1.85	CCB/ACEI/β-blocker
5	M/30	MHTN	230/170	Grade 4	42.57	148	167	1.61	0.49	181	2.76	CCB/ARB/β-blocker
6	M/61	MHTN	198/120	Grade 3	22.91	119	206	1.19	0.24	236	1.85	CCB/ACEI/α-blocker
7	M/31	MHTN	240/160	Grade 3	15.18	113	221	1.11	0.37	239	1.9	CCB/α-blocker/diuretics
8	M/40	MHTN	200/130	Grade 4	19.79	128	333	N.D.	N.D.	274	1.08	CCB/ACEI/β-blocker/α-blocker
Renal disease controls												
DC 1	M/40	MN	110/64	N.D.	152.85	141	223	N.D.	N.D.	194	5.56	ARB
DC 2	M/48	MN	103/69	N.D.	85.57	155	257	1.19	0.29	291	3.48	ARB
DC 3	M/45	MN	124/74	N.D.	89.78	143	362	1.27	0.35	167	3.93	ACEI
DC 4	F/36	LN-IV	97/59	N.D.	81.99	80	286	0.37	0.09	215	3.49	ACEI
DC 5	F/37	LN-V	114/88	N.D.	92.48	114	232	0.57	0.22	303	5.29	ARB
DC 6	M/35	IgAN-III	117/75	N.D.	89.01	148	195	N.D.	N.D.	158	0.64	ACEI
DC 7	M/28	IgAN-III	112/60	N.D.	98.48	162	206	N.D.	N.D.	220	0.38	ARB
DC 8	F/38	IgAN-III	150/100	Grade 1	139.34	131	304	1.10	0.24	171	0.84	ACEI
DC 9	M/20	IgAVN	146/95	Grade 1	127.49	124	274	1.04	0.22	166	0.74	ARB
DC 10	M/51	IgAVN	134/94	N.D.	59.97	112	228	1.32	0.34	189	7.63	ARB
DC 11	M/34	IgAVN	126/88	N.D.	101.09	132	245	1.18	0.28	225	2.25	ARB
Non-renal disease controls												
DC 12	M/66	DM	113/86	N.D.	111.19	123	124	N.D.	N.D.	247	Negative*	N
DC 13	F/64	DM	111/62	N.D.	74.01	124	127	N.D.	N.D.	288	Negative*	N
DC 14	M/65	DM	120/82	N.D.	81.31	163	195	N.D.	N.D.	175	Negative*	N
DC 15	F/76	DM	154/84	Normal	114.81	144	272	N.D.	N.D.	158	Negative*	CCB
DC 16	M/32	HTN	114/88	N.D.	141.19	154	217	N.D.	N.D.	356	N.D	N
DC 17	M/47	HTN	120/86	N.D.	127.07	146	244	N.D.	N.D.	253	N.D	N
DC 18	M/58	BD	142/71	N.D.	87.88	147	215	N.D.	N.D.	187	Negative*	ARB
DC 19	F/69	BD	110/72	N.D.	85.24	122	276	N.D.	N.D.	164	Negative*	ARB

*M* male, *F* female, *pMHTN* primary malignant hypertension, *MHTN* malignant hypertension, *MN* membranous nephropathy, *LN* lupus nephropathy, *IgAN* IgA nephropathy, *HTN*, hypertension, *BD* Behcet’s disease, *DM* diabetes mellitus, *N.D.* not done, *N* none, *CCB* calcium channel blocker, *ARB* angiotensin II receptor blocker; *ACEI* angiotensin-converting enzyme inhibitor. Hypertension-related retinopathy classification according to the classification of Keith–Wagener–Barker.

*Negative proteinuria by urinalysis.

**Figure 1:**
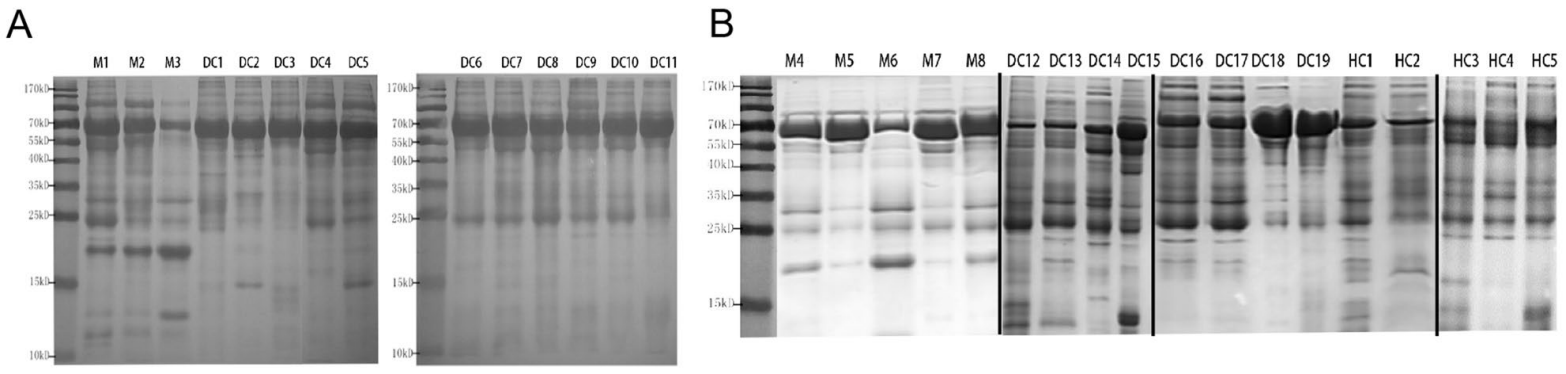
SDS-PAGE analysis of urine samples from different diseases. (**A**) A distinct band between 15 and 25 kDa was highly expressed in pMHTN patients (M1–3) compared to other renal diseases. (**B**) SDS-PAGE analysis involving pMHTN, non-renal DCs, and HCs revealed that the specific band was present in 4 out of 5 pMHTN patients (M4, 5, 6, 8), while it was absent in DCs and HCs.M1-M8: pMHTN; DC 1–3: membranous nephropathy; DC 4–5: lupus nephritis, DC 6–8: IgA nephropathy; DC 9–11: IgA vasculitis; DC12-15: diabetes mellitus; DC 16–17: hypertension; DC 18–19: Behcet’s disease; HC 1–5: Healthy control. The gel images were cropped for clarity and the full-length gels are presented in Supplementary Materials.

Through SDS-PAGE analysis, we identified a distinct band between 15 and 25 kDa that was highly expressed in pMHTN patients (M1–3) compared to other renal diseases. This band may represent a potential protein biomarker for pMHTN (Fig. 1A). Further SDS-PAGE analysis involving pMHTN, non-renal DCs, and HCs revealed that this specific band was present in 4 out of 5 pMHTN patients (M4, 5, 6, 8), while it was absent in DCs and HCs (Fig. 1B). The gel images were cropped for clarity and the full-length gels are presented in Supplementary Materials (Supplementary Fig. 1 & 2). Lanes corresponding to additional replicates of the experiment and samples from patients with ambiguous diagnoses were omitted, but without affecting the conclusions.

### LC–MS/MS identification of differential proteins

In the analysis of high-expression protein bands within 15–25 kD from three pMHTN patients (M1, M2, M3), in-gel digestion followed by MS identified 11 proteins. Compared to corresponding range bands in three HCs, a quantitative assessment using spectrum counting identified five differential proteins. The differential proteins identified include CFD, pancreatic ribonuclease, lithostathine-1-alpha, RBP4 and agrin (Table 2).

**Table 2:** The details of the differentially expressed proteins in pMHTN patients.

Protein name	Uniprot ID	MW (kDa)	Mass spectrum numbers						FC	p value
			HC1	HC 2	HC3	pMHTN1	pMHTN 2	pMHTN 3		
Complement factor D	P00746	27	0	0	0	7	11	14	∞	0.006
Pancreatic ribonuclease	P07998	18	1	0	0	17	11	15	43	0.001
Lithostathine-1-alpha	P05451	19	1	1	2	5	9	6	5	0.013
Retinol-binding protein 4	P02753	23	6	7	9	41	24	27	4.2	0.012
Agrin	O00468	215	1	1	0	3	2	3	4	0.013

*MW* molecular weight, *HC* healthy control, *pMHTN* primary malignant hypertension, *FC* fold change.

The p value represents the statistical difference in the mass spectrum numbers between pMHTN and HCs.

### ELISA validation of differential proteins

During the validation phase, we recruited 10 pMHTN patients, 37 DCs, and 30 HCs (Table 3). The disease control group consisted of 5 benign hypertensive nephrosclerosis, 13 IgA nephropathy (IgAN), 6 LN, and 13 MN. Among the 10 pMHTN patients, one presented with microangiopathic hemolytic anemia and thrombocytopenia, suggestive of MHTN with systemic TMA. The remaining patients had normal levels of hemoglobin, platelets, and LDH (Supplementary Table 2).

**Table 3:** Clinical characteristics of participants in the validation cohort before and after PSM.

Variables	Before matching				After matching		
	pMHTN (n = 10)	DC (n = 37)	HC (n = 30)	p	pMHTN (n = 8)	DC (n = 15)	p
Age, year	28.1 ± 9.1	42.3 ± 14.7*	40.6 ± 9.9^**#**^	**0.005**	27.0 ± 9.7	41.7 ± 11.2	**0.013**
Male, n (%)	7 (70)	18 (49)	19 (63)	0.238	4 (67)	8 (53)	0.659
BMI, kg/m^2^	23.22 ± 3.23	24.33 ± 3.74	21.13 ± 3.32	0.363	22.95 ± 3.93	25.25 ± 4.06	0.26
SBPmax, mmHg	208 (180, 228)	138 (120, 150)*	110 (104,122)^**#**^	** < 0.001**	199 ± 24	140 ± 18	** < 0.001**
DBPmax, mmHg	132 (123, 148)	90 (74, 94)*	82 (74,90)^**#**^	** < 0.001**	130 (123, 138)	90 (81, 97)	** < 0.001**
Scr, umol/L	171.00 (157.00, 213.00)	91.00 (65.00, 192.00)*	78.00 (73.25, 82.00)^**#**^	** < 0.001**	233.50 ± 113.56	186.67 ± 67.74	0.378
eGFR, mL/min/1.73m^2^	38.50 (31.00, 44.75)	77.00 (32.00, 99.00)*	102.00 (90.00, 109.75)^**#**^	** < 0.001**	32.00 (24.75, 36.25)	32.00 (27.00, 56.00)	0.876
glucose, mmol/L	4.74 ± 0.53	4.64 ± 0.78	4.72 ± 0.56	0.850	4.75 ± 0.64	4.86 ± 0.81	0.749
TG, mmol/L	1.74 (1.22, 3.11)	1.92 (1.56, 2.57)	1.54 (1.24, 1.78)	0.060	2.44 (1.64, 3.11)	1.95 (1.64, 2.62)	0.938
C3, g/L	0.96 ± 0.22	0.96 ± 0.24		0.917	1.00 ± 0.25	0.89 ± 0.25	0.379
C4, g/L	0.24 ± 0.10	0.22 ± 0.09		0.498	0.25 ± 0.11	0.24 ± 0.11	0.823
24hUP, g/24 h	0.96 (0.74, 1.49)	2.13 (1.15, 4.38)*		**0.015**	1.06 (0.96, 1.49)	3.14 (1.20, 5.22)	0.098

*PSM* propensity score matching, *pMHTN* primary malignant hypertension, *DC* disease control, *HC* healthy control, *BMI* body mass index, *SBP* systolic blood pressure, *DBP* diastolic blood pressure, *Scr* serum creatinine, *eGFR* estimated glomerular filtration rate, *TG* triglyceride, *24hUP* 24-h urinary protein.

*p < 0.05 pMHTN vs DC, ^#^p < 0.05 pMHTN vs HC.

Significant values are in bold.

**Figure 2:**
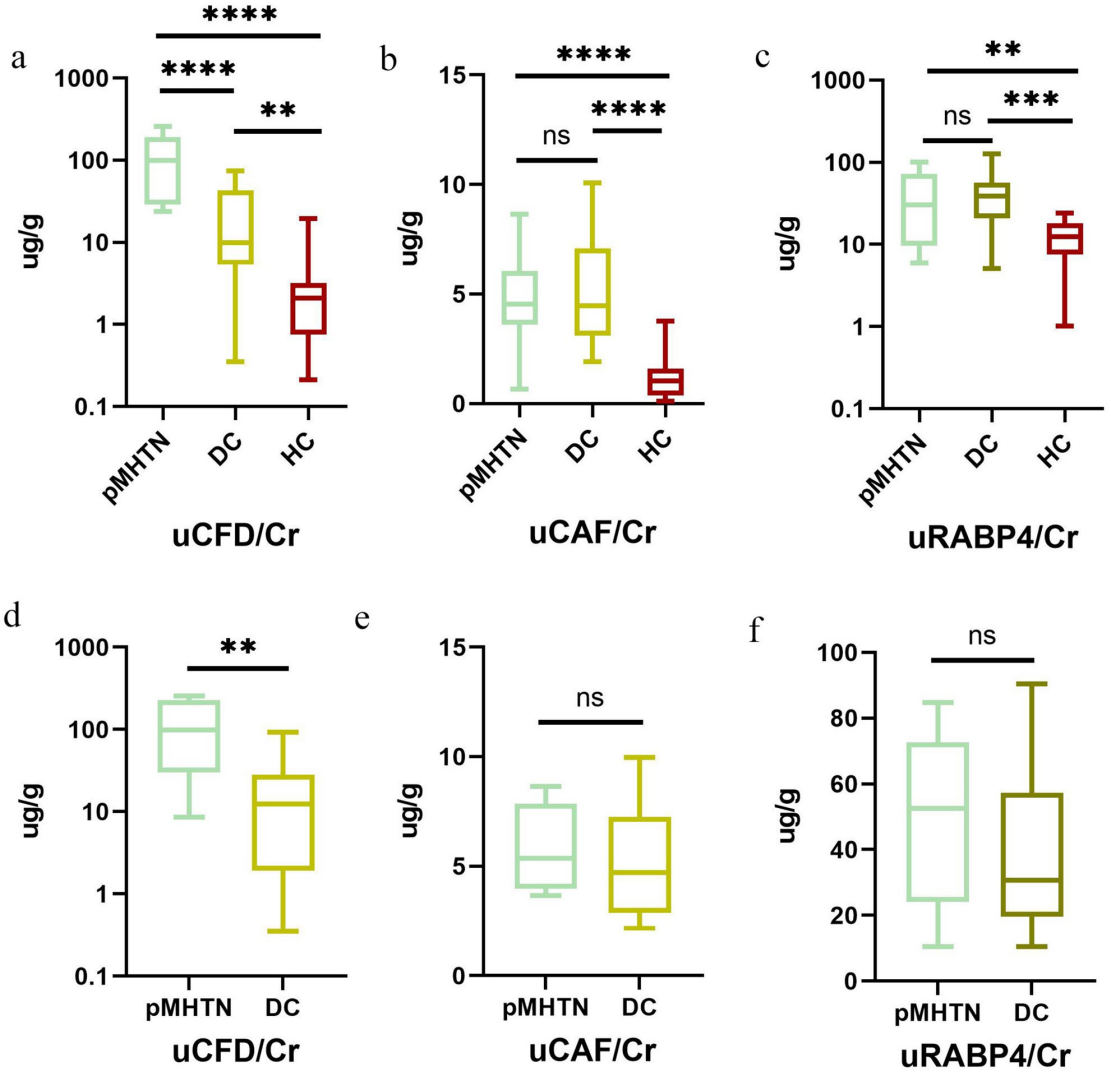
The expression of three differential proteins in the validation cohort before PSM (**a**-**c**). Amongst three urinary proteins, CFD/Cr exhibited significant differences, with the pMHTN group showing substantially higher values than both disease control and health control groups (p < 0.001). In contrast, there were no differences observed in urinary RBP4/Cr and CAF/Cr between the pMHTN group and disease controls group. The expression of three differential proteins in the validation cohort before PSM (**d**-**f**). The urinary CFD/Cr levels still revealed significant differences, whereas no statistical differences were found for RBP4/Cr and CAF/Cr.

We further validated the urinary levels of CFD/Cr, CAF/Cr, and RBP4/Cr using ELISA across three groups (Fig. 2). Amongst these urinary proteins, CFD/Cr exhibited significant differences, with the pMHTN group showing substantially higher values than both DC and HC groups (p < 0.001). In contrast, there were no differences observed in urinary RBP4/Cr and CAF/Cr between the pMHTN group and DC group (Fig. 2a–c).

To minimize bias arising from renal function and urinary protein levels, we employed the propensity score matching (PSM) to match the 24-hUP and eGFR between the pMHTN group and DC group. After matching, aside from age and blood pressure, there were no statistically significant differences in baseline data between the two groups (Table 3). After PSM analysis, the urinary CFD/Cr levels still revealed significant differences, whereas no statistical differences were found for RBP4/Cr and CAF/Cr (Fig. 2d–f).

### Diagnostic efficacy of urinary CFD/Cr for pMHTN

**Figure 3:**
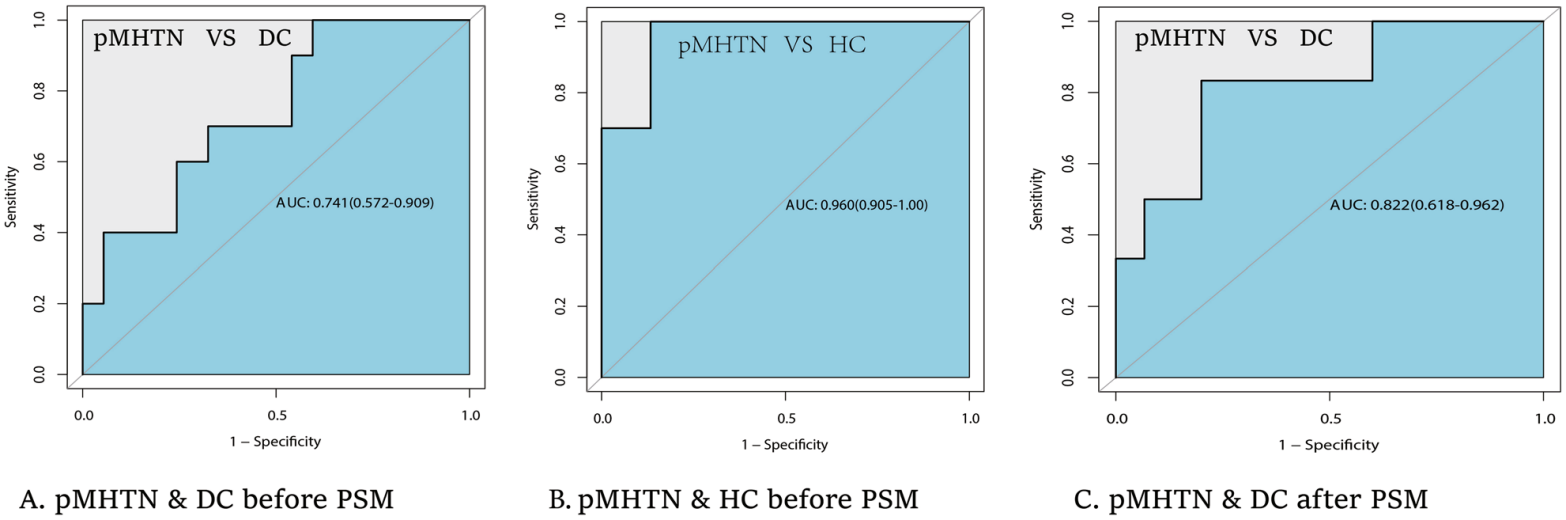
The ROC curves for urinary CFD/Cr for the diagnosis of pMHTN. ROC curves for urinary CFD/Cr for the diagnosis of pMHTN. (**A**) Prior to PSM, ROC curve analysis between pMHTN and DC group. The AUC for CFD/Cr was 0.741 (95% CI 0.572–0.909, p < 0.05). (**B**) Prior to PSM, ROC curve analysis between pMHTN and HC group. The AUC for CFD/Cr was 0.960 (95% CI 0.905–1.00, p < 0.001). (**C**) After PSM analysis, ROC curve analysis between pMHTN and DC group. The AUC for CFD/Cr was 0.822 (95% CI 0.618–0.962, p < 0.05).

The diagnostic efficacy of urinary CFD/Cr for pMHTN was assessed using the ROC curve (Fig. 3). Prior to PSM, the area under the curve (AUC) for CFD/Cr between the pMHTN group and DC group was 0.741 (95% CI 0.572–0.909, p < 0.05) (Fig. 3a), and it reached 0.960 (95% CI 0.905–1.00, p < 0.001) between the pMHTN group and HC group (Fig. 3b). After PSM analysis, the AUC for CFD/Cr between the pMHTN and DC groups was 0.822 (95% CI 0.618–0.962, p < 0.05), with a sensitivity of 0.833 and a specificity of 0.80 (Fig. 3c).

## Discussion

pMHTN is a renal emergency that can lead to deterioration of kidney function, and there is a lack of non-invasive biomarkers to improve diagnosis and unravel its pathophysiological mechanisms. In this study, we found significantly elevated levels of urinary CFD in biopsy-proven pMHTN patients through SDS-PAGE and LC–MS/MS analysis. The validation analysis revealed that urinary CFD levels were markedly increased in the pMHTN group compared to those with IgAN, MN, LN, and benign hypertensive nephrosclerosis. These data suggest the potential of urinary CFD as a diagnostic biomarker for pMHTN and indicate the involvement of the alternative complement pathway activation in the pathogenesis of pMHTN.

Past research considered shear stress-induced endothelial damage as the main contributor to renal injury in MHTN. However, recent studies have indicated that complement activation, inflammation, and oxidative stress could also cause microvascular endothelial damage^15^. In particular, the activation of the alternative pathway (AP) may play a critical role in renal injury of pMHTN^16–18^. Timmermans et al. reported patients with primary severe hypertension accompanied by renal TMA who exhibited mutations in genes encoding for complement components. The activation of the complement system was evidenced by increased plasma levels of soluble C5b-9 and deposition of C4d, C3c, and C5b-9 on renal vascular and glomerular capillary walls, suggesting that severe hypertension could trigger complement activation, leading to the occurrence of TMA^17^. Additionally, serum samples collected at diagnosis from these patients induced abnormal C5b-9 formation on microvascular endothelium, reflecting active complement dysregulation^16^. However, these studies did not evaluate urinary AP-related complement levels. Zhang et al. assessed AP pathway-associated complements in the urine and plasm between pMHTN and healthy controls, and found significantly higher urinary CFD levels in pMHTN patients, aligning with our findings^18^. However, this study only compared pMHTN patients with a healthy control group, lacking an analysis against other kidney diseases, thus not assessing the potential diagnostic value of urinary CFD for pMHTN. Our study, by comparing pMHTN with various disease controls, firstly underscored the diagnostic value of urinary CFD for pMHTN.

CFD is a 24 kDa serine protease consisting of 228 amino acids, with a normal blood concentration of approximately 1–2 ug/mL^19^. CFD is involved in the initiation and regulation of the AP, acting as a critical rate-limiting enzyme for this pathway^19^. CFD is completely reabsorbed in the renal tubules following glomerular filtration and is rapidly degraded at the intracellular level. Kidneys regulate blood CFD concentration through the glomerular filtration rate^20^. Given that both serum and urine CFD levels are significantly elevated in patients with renal insufficiency^21,22^, we matched the eGFR of the pMHTN group with the DC group, and urinary CFD/Cr levels were approximately tenfold higher in the pMHTN group compared to DC group after PSM. The elevated urinary CFD levels in pMHTN may be related to systemic dysregulation of the AP or local complement activation within the kidneys. Nonetheless, there is a distinct possibility that hypertension may be provoked by complement abnormalities, which in turn aggravate hypertension and result in positive feedback dysregulation of the complement system^23^. Cavero et al.^24^ provide additional persuasive evidence regarding the fundamental role of the complement system in hypertension-associated TMA. In their cohort of 55 atypical hemolytic uremic syndrome (aHUS) patients, 36 exhibited grade II or III hypertension while 19 showed grade III/IV retinopathy. Genetic complement abnormalities existed in 37% of the MHTN group. Antihypertensives were administered to all patients but only one exhibited hematological and renal responses. Eculizumab treatment resulted in renal and hematological responses in 7 of 9 MHTN patients. In our two pMHTN cohorts, only one patient had hypertension-associated TMA. It remains unclear whether complement dysregulation is present in our pMHTN patients.

Several small-molecule CFD inhibitors have entered clinical trials for treating diseases mediated by the AP, such as C3 glomerulopathy, and aHUS^19^. Although effective blood pressure management in MHTN can lead to favorable renal and overall outcomes, a subset of patients continued disease progression^3,5^. Our study, alongside existing data, suggested CFD inhibitors may serve as a targeted treatment for pMHTN, especially when complicated by TMA. Future research should investigate the dysregulation of complement activity in pMHTN as a potential therapeutic direction.

Through LC–MS/MS analysis, we also identified other two differential proteins: Lithostathine-1-alpha and Pancreatic ribonuclease. Lithostathine-1-alpha is predominantly secreted by the exocrine pancreas and may play a role in pancreatic stone formation and the pathogenesis of diabetes mellitus^25,26^. Pancreatic ribonuclease, which participates in RNA cleavage, is a pyrimidine-specific endoribonuclease highly expressed in pancreatic tissue^27^. To our knowledge, there are no reports linking these two proteins with kidney diseases. In an independent cohort, we validated RBP4 and the C-Terminal Fragment of Agrin. Urinary RBP4 is a biomarker for proximal tubular renal disease^28^. Agrin is a type of heparan sulfate proteoglycan that constitutes an essential part of the glomerular basement membrane and extracellular matrix^29^. With a molecular weight of 215 kDa, agrin does not conform to the molecular weight range of differential bands, however, the CAF is a product of Agrin cleavage by proteases, specifically with a molecular weight of 22 kDa. CAF is filtered by the glomeruli and reabsorbed in the renal tubules^30^. Our results indicate no significant differences in the levels of urine RBP4 and CAF between the pMHTN group and the DC group, suggesting that the diagnostic specificity of these two proteins for pMHTN is limited.

One limitation of our study is the small number of patients included, and its cross-sectional design, which precludes determining a causal relationship between CFD and pMHTN. Moreover, our study did not assess the urinary CFD levels in patients with secondary MHTN. There is also a lack of validation of CFD deposition in renal tissue. Thirdly, the pMHTN patients were younger than the control groups in the validation cohort. The potential influence of age on urinary CFD levels cannot be ruled out. Future large-scale longitudinal cohort studies are warranted to verify the role of CFD in pMHTN and to explore the relationship between urinary CFD levels and disease progression in pMHTN.

## Conclusion

We report for the first time that the levels of urinary CFD/Cr are significantly elevated in biopsy-proven pMHTN patients and can serve as a potential diagnostic biomarker for pMHTN. There is a need for prospective studies with larger samples to validate this finding and explore the role of CFD in the pathogenesis and progression of pMHTN. Since pMHTN may involve the activation of AP, CFD inhibitors may be a potential therapeutic option for some patients with pMHTN.

## Methods

### Participants and study design

The study was conducted in two phases. In the first phase, we selected all biopsy-proven pMHTN patients from Peking Union Medical College Hospital and had complete clinical and pathological data between July 2013 and July 2014. Concurrently, hospitalized patients with other diseases during the same period were randomly chosen as the DCs), alongside healthy individuals as HCs. Urinary SDS-PAGE analysis revealed differential bands, and the proteins within these bands were subsequently digested with in-gel proteases and identified using two-dimensional LC–MS/MS.

**Figure 4:**
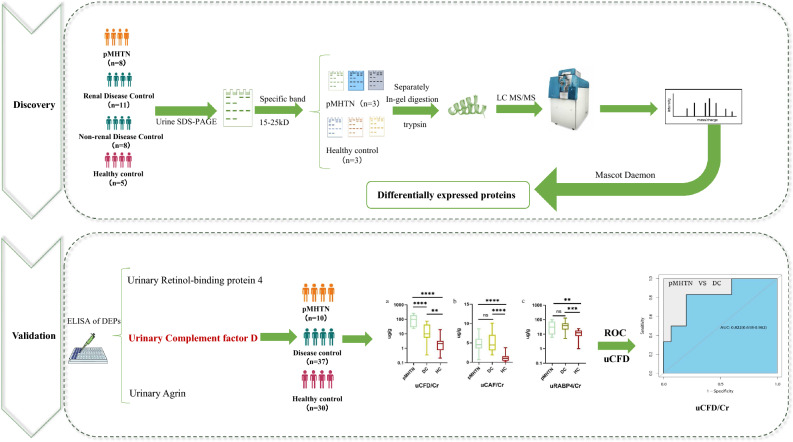
Flowchart of the study. *pMHTN* primary malignant hypertension, *SDS-PAGE* sodium sodecy sulfate polyacrylamide gel electrophoresis, *LC–MS/MS* liquid chromatography-tandem mass spectrometry, *ELISA* enzyme-linked immunosorbent assay, *DEPs* differentially expressed proteins, *CFD* complementary factor D, *CAF* C-terminal Agrin fragment, *RBP4* retinol-binding protein 4, *ROC* receiver operating characteristic.

In the second phase, we prospectively recruited all patients with biopsy-proven pMHTN at our hospital between June 2017 and September 2018. As the DCs, patients with other renal diseases diagnosed by renal biopsy from June 2018 to September 2018 were included. The differential proteins identified by LC–MS/MS were validated using the ELISA method (Fig. 4).

The diagnosis of pMHTN must concurrently meet the following criteria: (1) Severe hypertension: diastolic blood pressure > 120 mmHg and/or systolic blood pressure > 180 mmHg on multiple measurements; (2) Fundoscopic examination: bilateral retinal arteriolar blot- or flame-shaped hemorrhages, or “cotton wool” exudates, possibly accompanied by papilledema (Keith–Wagener classification of Grade III or IV); (3) Renal pathology: characteristic histopathological changes of malignant nephrosclerosis, including intimal thickening and fibrosis with luminal narrowing of the interlobular and afferent arterioles, concentric myointimal proliferation (‘onion skin’ lesions), possibly with fibrinoid necrosis or thrombosis. Pathological examination of renal tissues included light microscopy (Hematoxylin & Eosin, Periodic Acid-Schiff, Periodic Acid-Silver Methenamine, and Masson’s Trichrome staining), electron microscopy, and immunofluorescence for IgG, IgM, IgA, C3, C4, C1q, fibrinogen, HBsAg, and HBcAg. The renal biopsy specimens were independently assessed by two nephropathologists. Exclusion criteria included secondary hypertension, such as renal parenchymal and renovascular hypertension and endocrine-related hypertension. Endocrine-related secondary hypertension was investigated with measurements of plasma renin/angiotensin II/aldosterone levels in supine and upright positions, thyroid hormones, serum ACTH, 24-h urinary catecholamines, and 24-h urinary free cortisol levels.

We collected various clinical and laboratory parameters, including age, gender, body mass index, blood pressure, hemoglobin levels, platelet count, complement C3 and C4 levels, lactate dehydrogenase (LDH), 24-h urinary protein (24hUP), serum creatinine, estimated glomerular filtration rate (eGFR), blood glucose, lipid profiles, and types of antihypertensive medications administered. The eGFR was assessed using the Chronic Kidney Disease Epidemiology Collaboration (CKD-EPI) equation.

### Urine collection and preservation

Midstream urine samples (30 mL) were collected from patients on the morning of renal biopsy. The samples underwent centrifugation at 3000 rpm for 10 min, followed by filtration through nitrocellulose membranes to adsorb urinary proteins. The membranes were dried and stored at − 80 °C in vacuum-sealed bags.

### Urine SDS-PAGE and in-gel digestion

Urine proteins preserved on nitrocellulose membranes were eluted by heating and protein concentrations were measured using the Bradford method. The eluted proteins were mixed with PAGE sample buffer (50 mM Tris–HCl, pH 6.8 with 50 mM DTT, 0.5% SDS and 10% glycerol), heated at 95 °C for 5 min, and then separated by SDS-PAGE using 12% acrylamide gels. After electrophoresis, the differential bands between 15 and 25 kD were excised from gels from both pMHTN patients and HCs. A detailed description of the in-gel digestion can be found in Supplemental Material. Briefly, the gel fragments were washed, incubated, reduced, alkylated and digested. The extracted peptide solution from each patients was freeze-dried under vacuum and analyzed separately by LC–MS/MS.

### LC–MS/MS analysis

Lyophilized peptides were redissolved in 0.1% formic acid and subjected to chromatography using a Waters Ultra-performance LC system. Peptides were separated on a 10 cm fused silica column packed in-house using ReproSilPur C18-AQ (3 µm resin). Analysis of eluted peptides was performed using Triple TOF 5600, operating in positive ion mode, with a gradient elusion process and the LC–MS/MS settings are detailed in Supplementary Material.

### Data processing and protein quantitation

All MS/MS results were analyzed using Mascot software (Matrix Science, version 2.4.0) searching against the SwissProt human database (http://www.uniprot.org/). Search criteria included: fixed modification of carbamidomethyl cysteine; variable modifications included methionine oxidation and protein N-terminal acetylation. Trypsin (cleavage at K, R) was used as the enzyme definition, allowing two missed cleavages site. For the data of Triple TOF 5600, the mass tolerances in MS and MS/MS were both set to 0.05 Da.

Scaffold software (version 4.0.1, Proteome Software Inc., Portland, OR, USA) was used for label-free quantitation analysis based on peptide-spectrum matching using data from MASCOT 2.4.0. Search conditions included: peptide identification confidence level of ≥ 90.0%, false discovery rate (FDR) ≤ 0.1%; protein identification was confirmed with FDR ≤ 0.1% and at least two identified peptides per protein. Identified urinary proteins were quantified based on mass spectrum counts. Proteins with a fold change greater than 2 and a p-value of less than 0.05 were selected as differential proteins.

### ELISA analysis

Differential proteins were validated using ELISA according to the manufacturer’s instructions. Urinary CFD was measured with a human ELISA Kit (R&D Systems, Minneapolis, MN, USA). Urinary Retinol-binding protein 4 (RBP4) was detected by human ELISA Kit (Abcam, Cambridge, MA, USA; Immunoway, Plano, TX, USA). Urinary Agrin was identified by human C-terminal Agrin fragment (CAF) ELISA Kit (Mlbio, Shanghai, China). All urinary proteins were normalized against urinary creatinine levels.

### Statistical analysis

Normally distributed quantitative data were expressed as mean ± standard deviation, and comparisons between two groups were performed using independent sample t-tests, while analysis of variance (ANOVA) was employed for comparisons among multiple groups. Non-normally distributed quantitative data were expressed as the median and interquartile range, with non-parametric analyses for group comparisons. Categorical variables were expressed as case numbers and percentages, with chi-square tests conducted for inter-group comparisons. During the validation phase, propensity score matching (PSM) was employed to reduce the effects of confounding factors such as eGFR and 24-hUP, using the nearest-neighbor matching algorithm (caliper width 0.2 of the standard deviation of the logit score). Receiver operating characteristic (ROC) curves were generated to determine the diagnostic performance of the differential proteins. A p-value of < 0.05 was considered statistically significant. Due to the lack of previous studies, we did a post hoc power calculation to evaluate the statistical power of current study (n = 8 pMHTN patients/15 disease controls). Based on the means and standard deviations of urinary CFD/Cr between the two groups after propensity score matching, at a significance level of α = 0.05, the statistical power was calculated to be 82.97%^31^. All data analyses were performed using Graphpad Prism 8.0 and R, version 4.0.2.

### Ethical approval and informed consent

This study was conducted in accordance with the Declaration of Helsinki and approved by the Ethics Committee of Peking Union Medical College Hospital (JS1233-1). All participants provided written informed consent after being informed of the study’s purpose.

### Supplementary Information

Supplementary Information.

## Data Availability

The data that support the findings of this study are available from the corresponding author upon reasonable request.
