# Supplementary material for: Urinary complement factor D is increased in primary malignant hypertension: a single-center, cross-sectional study
Source: Sci Rep. 2024 Jul 15;14:16253. doi: 10.1038/s41598-024-66875-4 (PMC11251191; doi:10.1038/s41598-024-66875-4)
Supplement: Supplementary file 1 — Supplementary Information. [file 41598_2024_66875_MOESM1_ESM.pdf]

Urinary complement factor D is increased in primary malignant hypertension: a single-center, cross-sectional study

Yaqi Cheng[1,#], Weiwei Qin[2,3,#], Liling Lin[4], Youhe Gao[3,*], Mingxi Li[1,*]

1. Department of Nephrology, State Key Laboratory of Complex Severe and Rare Diseases, Peking Union Medical College Hospital, Peking Union Medical College and Chinese Academy of Medical Sciences, Beijing 100730, China.
2. Department of Anesthesiology, Qingdao Hospital, University of Health and Rehabilitation Sciences (Qingdao Municipal Hospital), Qingdao 266071, China.
3. Beijing Key Laboratory of Gene Engineering Drug and Biotechnology, College of Life Sciences, Beijing Normal University, Beijing 100875, China.
4. Department of Laboratory, State Key Laboratory of Complex Severe and Rare Diseases, Peking Union Medical College Hospital, Peking Union Medical College and Chinese Academy of Medical Sciences, Beijing 100730, China.

# Authors contributed equally.

*** Corresponding author**:

Mingxi Li[1*]

Department of Nephrology, State Key Laboratory of Complex Severe and Rare Diseases, Peking Union Medical College Hospital, Chinese Academy of Medical Science and Peking Union Medical College, Beijing, China.

No.1 Shuaifuyuan Street, Beijing, 100730, China

Tel: 8610-65295058

mail: mingxili@hotmail.com

Youhe Gao[3,*]

Beijing Key Laboratory of Gene Engineering Drug and Biotechnology, College of Life Sciences, Beijing Normal University, Beijing, China.

No.19, Xinjiekouwai Street, Haidian District, Beijing, 100875, China.
Tel：8610-58804382
E-mail: gaoyouhe@bnu.edu.cn

Supplementary methods

In-gel digestion

The gel fragments were washed twice at 37°C with 100 mM ammonium bicarbonate solution for 15 minutes; then incubated with 100 mM ammonium bicarbonate/acetonitrile (1:1) at 37°C for 15 minutes. After cooling to room temperature, samples were reduced with 20 mM dithiothreitol at 56°C for 1 hour, then alkylated with 55 mM iodoacetamide in the dark at room temperature for 45 min. The dried gel pieces were digested overnight with trypsin at a protease: substrate ratio of 30:1 at 37°C. The supernatant was collected and the gel pieces were further extracted twice with 50% acetonitrile/5% formic acid for 60 minutes each to extract peptides. The extracted peptide solution was freeze-dried under vacuum.

LC-MS/MS analysis

The eluted peptides were analyzed by a Triple TOF 5600. Elution was performed over a gradient of 5–28% buffer B (0.1% formic acid, 99.9% ACN; flow rate, 0.3 μL/min) for 60 min. The MS data were acquired using an ion spray voltage of 3kV, curtain gas of 20 PSI, nebulizer gas of 30 PSI, and an interface heater temperature of 150°C. The precursors were acquired in 500 ms ranging from 350 to 1250 m/z, and the product ion scans were acquired in 50 ms ranging from 250 to 1800 m/z. A rolling collision energy setting was used. A total of 30 product ion scans were collected if exceeding a threshold of 125 counts per second (counts/s) and with a +2 to +5 charge state for each cycle.

Supplementary tables

Supplementary Table 1. Comparison of clinical data between pMHTN and DC groups in the discovery phase

Variables	pMHTN (n = 8)	DC (n = 19)	p
Age, year	36 (31, 42)	47 (37, 64)	**0.075**
Male, n (%)	8(100)	10 (59)	0.057
SBPmax, mmHg	217 (200, 230)	114 (111, 124)	**< 0.001**
DBPmax, mmHg	143 (130, 152.5)	75 (64, 87)	**< 0.001**
Scr, umol/L	286 (197, 344)	74 (66, 91)	**< 0.001**
eGFR, ml/min/1.73m^2	26.32±10.94	102.10±25.38	**< 0.001**
hemoglobin, g/L	133.50 ± 15.09	135.24 ± 22.21	0.821
platelet, ×10^9/L	235.50 ± 52.55	237.94 ± 61.99	0.92
LDH, U/L	194.50 (179.75, 236.75)	215.00 (171.00, 253.00)	0.749

Abbreviation: pMHTN, primary malignant hypertension; DC, disease control; HC, healthy control; SBP, systolic blood pressure; DBP, diastolic blood pressure; Scr, serum creatinine; eGFR, estimated glomerular filtration rate; LDH, lactate dehydrogenase.

Supplementary Table 2. Clinical features and laboratory findings of pMHTN patients in validation phase.

Number	Gender /Age	Diagnosis	BP, mmHg	Hypertensive Retinopathy	eGFR, ml/min/1.73m^2	Hb, g/L	Plt, ×10^9/L	LDH， U/L	C3, g/L	C4, g/L	24hUP, g	Antihypertensive drugs
1	M/25	MHT	240/170	Grade 4	71.99	156	180	204	0.99	0.28	1.06	CCB/ACEI/α-blocker /β-blocker
2	M/25	MHT	230/150	Grade 4	53.25	148	248	161	0.75	0.16	0.78	CCB/ACEI/β-blocker
3	F/18	MHT	180/130	Grade 4	18.38	122	583	269	1.16	0.24	0.96	CCB/ARB/β-blocker
4	M/33	MHT	180/140	Grade 4	33.44	130	258	208	1.35	0.25	0.62	CCB/ACEI/α-blocker
5	M/28	MHT	200/100	Grade 3	47.27	136	182	214	0.98	0.21	0.82	CCB/ARB/β-blocker
6	M/31	MHT	270/160	Grade 4	36.34	119	229	174	1.07	0.32	0.30	CCB/ARB/β-blocker
7	M/42	MHT	180/120	Grade 3	40.49	152	205	143	0.83	0.21	0.74	CCB/ARB/ diuretics
8	F/42	MHT	215/120	Grade 4	32.52	87	207	284	0.95	0.43	1.49	ARB/β-blocker/CCB
9	M/16	MHT	180/130	Grade 3	16.58	107	80	823	0.60	0.09	2.05	CCB/ACEI/α-blocker /β-blocker
10	F/21	MHT	220/133	Grade 3	44.02	112	195	185	N.D.	N.D.	1.78	CCB/ARB/α-blocker

Abbreviation: M, male; F, female; MHT, malignant hypertension; N.D.., not done; CCB, calcium channel blocker; ARB, angiotensin II receptor blocker; ACEI, angiotensin-converting enzyme inhibitor. Hypertension-related retinopathy classification according to the classification of Keith-Wagener-Barker

Supplementary figure

Supplementary Figure 1 The Full-length gel images of repeated experiments for Figure 2A.

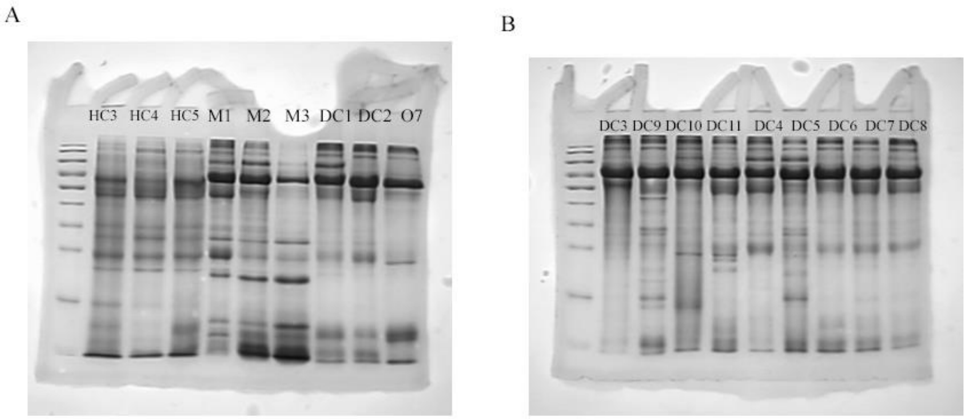

During the discovery phase of urine SDS-PAGE experiments, we conducted exploratory experiments on patients M1-3 and DC1-11, as shown in Figure 2A. To validate the experimental results, we performed repeat analyses on samples from these patients (M1-3 and DC1-11). The repeat SDS-PAGE analysis was conducted alongside healthy controls (HC3/4/5) and another sample (O7, which was excluded from subsequent analysis due to an ambiguous diagnosis), where HC3/4/5 and O7 were the initial subjects of SDS-PAGE analysis. As the original experiments were conducted some time ago, we are providing the complete gel images of the repeat experiments for M1-3 and DC1-11 as Supplementary Figure 1; where Supplementary Figure 1 (A) corresponds to Supplementary Figure 2 (D) as the original gel image for HC3/4/5. The results of the repeat experiments for these patients are consistent with the initial analysis (Figure 2A), supporting our initial findings.

(1) Supplementary Figure 1 (A) includes the complete gel images of the repeat analysis for patients M1-M3 and DC1-DC2. In the case of 3 pMHTN patients (M1, 2, 3), there is also a distinct band between 15-25 kDa.

(2) Supplementary Figure 1 (B) contains the complete gel images of the repeat analysis for patients DC3-DC11.

M1-M3: pMHTN; DC1-3: Membranous nephropathy; DC4-5: Lupus nephritis; DC6-8: IgA nephropathy; DC9-11: IgA vasculitis. HC3-5: Original gel images of healthy controls in Figure 2B. O7 was excluded from analysis due to an ambiguous diagnosis.

Supplementary Figure 2 The original gel images used in Figure 2B.

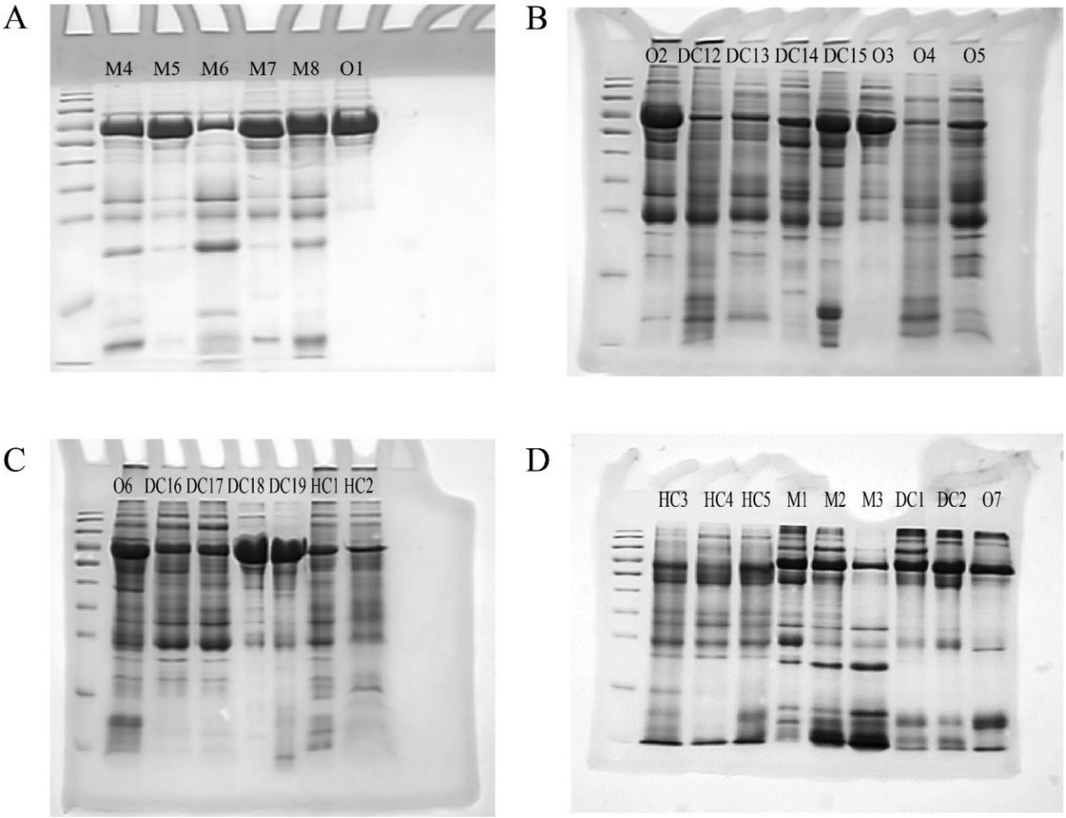

(A) A distinct band between 15-25 kDa was highly expressed in in 4 out of 5 pMHTN patients (M4, 5, 6, 8), O1 was omitted due to ambiguous diagnoses. (B) SDS-PAGE analysis involving non-renal DCs revealed that the specific band was absent in DCs, O2-5 were omitted due to ambiguous diagnoses. (C) SDS-PAGE analysis involving non-renal DCs and HCs revealed that the specific band was absent in DCs and HCs, O6 was omitted due to ambiguous diagnoses. (D) HC3/4/5 and O7 were included in the SDS-PAGE analysis along with the repeated samples from M1-3 and DC1-2. O7 was excluded from analysis due to an ambiguous diagnosis.M1-M8: pMHTN; DC 1-2: membranous nephropathy; DC12-15: diabetes mellitus; DC 16-17: hypertension; DC 18-19: Behcet's disease; HC 1-5: Healthy control.
